# A Reinforcement Learning Framework for Spiking Networks with Dynamic Synapses

**DOI:** 10.1155/2011/869348

**Published:** 2011-10-23

**Authors:** Karim El-Laithy, Martin Bogdan

**Affiliations:** Department of Computer Engineering, Faculty of Mathematics and Computer Science, Johannisgasse 26, 04103 Leipzig, Germany

## Abstract

An integration of both the Hebbian-based and reinforcement learning (RL) rules is presented for dynamic synapses. The proposed framework permits the Hebbian rule to update the hidden synaptic model parameters regulating the synaptic response rather than the synaptic weights. This is performed using both the value and the sign of the temporal difference in the reward signal after each trial. Applying this framework, a spiking network with spike-timing-dependent synapses is tested to learn the exclusive-OR computation on a temporally coded basis. Reward values are calculated with the distance between the output spike train of the network and a reference target one. Results show that the network is able to capture the required dynamics and that the proposed framework can reveal indeed an integrated version of Hebbian and RL. The proposed framework is tractable and less computationally expensive. The framework is applicable to a wide class of synaptic models and is not restricted to the used neural representation. This generality, along with the reported results, supports adopting the introduced approach to benefit from the biologically plausible synaptic models in a wide range of intuitive signal processing.

## 1. Introduction

Learning in neural networks can be achieved by two main strategies, namely, supervised and unsupervised learning. Unsupervised learning is guided by correlations in the input information to the network. Donald Hebb postulated in 1949 [20] that the modifications in the synaptic transmission efficacy are driven by the correlations in the firing activity of the pre- and postsynaptic neurons. Spike-timing-dependent plasticity (STDP) is the potentiation of a synapse when the postsynaptic spike follows the presynaptic spike within a time window of a few tens of milliseconds and the depression of the synapse when the order of the spikes is reversed. Since this is consistent with the postulates of D. Hebb, sometimes this type of STDP is referred to as Hebbian STDP. When the sign of the change in the synaptic strength is changed, the process might be known as anti-Hebbian STDP [17]. The Hebbian learning rules implement this dependence of synaptic changes on the relative timing of pre- and postsynaptic action potentials, and the Hebbian modulation of STDP is the synaptic changes following a learning algorithm either via the Hebbian or the anti-Hebbian rule [19]. One of the attractive models in this regard is the Bienenstock-Cooper-Munro (BCM) model for the development of orientation selective cells in the visual system [4]. The Hebbian learning rule of this model has received considerable support from experiments on long-term potentiation (LTP) and long-term depression (LTD) [31].

Many studies have investigated how the Hebbian-based learning algorithms can be applied to empower the performance of artificial neural networks (ANNs) and especially of those use either spiking neuronal models and/or synaptic models that implement STDP; see, for example, [15, 37] for recent reviews. A correlation-based Hebbian learning rule for spiking neurons was presented reporting that correlations between input and output discharges tend to stabilize [21]. A biologically plausible learning algorithm for multilayer neural networks was introduced in [23]. It was shown that the learning algorithm has allowed the network to solve partially the exclusive-Or (XOR) problem without back propagation. Applying both Hebbian and anti-Hebbian rules in a recurrent network that implements STDP was investigated [7]. It has been shown that leads to approximate convergence of the synaptic weights. These studies were focused on the computational properties of STDP, thus they have illustrated its function in neural homeostasis and supervised and unsupervised learning.

Notably, a number of theoretical analyses have reported that Hebbian and anti-Hebbian modulation of STDP can either minimize or maximize the postsynaptic (neuronal) firing variability to a given specific presynaptic input [7, 17, 39]. These studies have suggested that combining Hebbian rules and reinforcement learning (RL) [34] facilitates the simulation of the learning abilities featuring biological neural systems. A number of studies have investigated the tenability of integration between both concepts. For example, the ability to reduce the required learning steps for certain tasks in comparison to applying RL alone was investigated [5]. The tasks were poorly defined to be used for general machine learning regimes. Applying the RL rules to the spike-response model (SRM) was performed [11]. This has been done by adding a Hebbian term to the RL rule. The latter study was directed as well to investigate the influence on the number of learning steps. It has been showed that RL can occur via correlating the fluctuations in irregular spiking with a reward signal in networks composed of neurons firing Poisson spike trains [33, 40]. Another study has tried to teach a network of spiking neurons to output specific firing patterns on different time scales and in response to varying input combinations [15].

Commonly through all these studies, the modulation targets solely the synaptic weights in the synaptic parametrization, that is, only the spike-timing *independent* part of the synaptic parametrization is tuned. Little attention has been paid to the direct modulation of the synaptic hidden parameters, for example, response and recovery time constants.

In order to get an impression about the relevance of applying a learning rule to tune directly the synaptic hidden parameters, some topics are reviewed in the following. Adopting the spike-timing dependency in the synaptic action presumes that pre- and postsynaptic spiking activities influence the internal mechanisms result in the synaptic action itself. Shortly stated, there is a sort of closed-loop feedback mechanism regulating the synaptic action observed through changes in the synaptic plasticity [28, 41]. In chemical synapses, the calcium ions buffering plays, in general, a facilitatory role and is triggered by arriving spikes at the presynaptic terminal. This buffering enhances the transmission of the presynaptic spike by urging the release of neurotransmitter from the vesicles into the synaptic cleft. The extent of this facilitatory role is, however, bound to the contribution of other mechanisms such as the pool size of the ready to release vesicles and postrelease recovery timing constants of neurotransmitter. There is a dependence between the utilization of the synaptic resources (ions and neurotransmitter), and the overall synaptic action is modulated by the spike timing at the presynaptic site. The synaptic action consequently affects the postsynaptic activity. Latencies between postsynaptic spikes allow for the uptake of neurotransmitter from the cleft and for the reformation of vesicles within the presynaptic terminal. These latencies are basically modulated by the release process that is originally presynaptically regulated [41]. Thus, there is an interdependence between STDP (as the correlation between presynaptic and postsynaptic spiking) and the synaptic resources, for example, the concentration of neurotransmitter and ions. As briefed, the interdependence originates from the relation between the synaptic action and the relative timing of pre- and postsynaptic action potentials. This interdependence suggests that learning frameworks, in general, may specifically tune the internal synaptic dynamic mechanisms according to predefined inputs/outputs combinations.

For the class of synaptic models that implement STDP, the overall synaptic response originates from two contributions: the synaptic weight and the dynamic spike-timing dependent mechanisms. The latter arises from the synergy among the hidden synaptic parameters for example, via response time constants and scaling factors. Maass and Zador have reported that applying gradient descent tuning to hidden parameters of their stochastic synaptic model can lead in principle to learning within a neural circuit [27, 30]. This approach is based on the previous work by [1, 2, 29]; it has been shown that synaptic dynamics modelled, in general, as finite-impulse response filters can be learned through modulating their hidden parameters. Biologically plausible synaptic models that implement temporal coding via STDP can be characterized in general as integrated (multilayered) finite-impulse response filters [18].

It is tempting, therefore, to investigate whether the Hebbian/anti-Hebbian modulation of STDP within an RL framework, that is, with a reward signal, can lead to RL when the learning is directed to tune the hidden parameters of a synaptic model. In the study at hand, we propose a follow-up study to the introductory framework introduced in [12]. (The results reported here are separately produced and not adopted from [12].) The framework integrates the concepts of both Hebbian/anti-Hebbian learning and RL while explicitly using plausible biological neuronal and synaptic representations. The introduced training algorithm affect the values governing the synaptic dynamics (for example, time constants) instead of changing the synaptic weight. To illustrate this, the learning of the exclusive-OR (XOR) computation has been chosen. The simulated spiking neural network uses (a) Markram-Tsodyks synaptic model [28], and (b) Leaky integrate-and-fire neurons. The proposed approach is inspired from the learning algorithm for stochastic synapses that was introduced in [13]. Up to the knowledge of the authors, this is the first trial to develop such a framework to train the hidden synaptic parameters in a dynamic synaptic model.

It is not intended to introduce a novel network-based solution for the XOR problem; rather, the XOR task is chosen as a classic benchmark problem for learning algorithms. The core objective is to propose an appropriate, but yet simple, learning algorithm that implements both Hebbian and RL rules for spiking networks with spike-timing-dependent synapses via tuning the synaptic model parameters rather than the synaptic weights. The availability of such a framework opens new avenues in adopting the class of biophysical synaptic models in processing of neural signals and computations. Some of these synaptic models do not feature any scalar weight factors as synaptic weights (see, for example, [13, 24]), which is why they are not utilized widely in signal processing tasks that require the tuning of model parameters to achieve certain regime of dynamics characterized by predefined mapping between input and output spike patterns.

## 2. Models

**Neuronal Model**

Neurons are modelled as leaky integrate-and-fire (LIaF) neurons [6]. Each neuron is described by its voltage membrane potential *V*

(1) $$\begin{array}{c} \tau_{V}\frac{dV\left(t\right)}{dt}=V_{\text{rest}}-V\left(t\right)+\text{EPSP}\left(t\right), \end{array}$$

where *τ*_*V*_ is the membrane time constant set at 20 msec and EPSP is the total observed excitatory postsynaptic potential from all presynaptic terminals. When *V*(*t*) ≥ *V*_th_, a spike is generated and *V*(*t* ^+^): = *V*_rest_, where *t* ^+^ is the time instant after *t* and *V*_rest_ = 0 mV and *V*_th_ = 50 mV. An absolute refractory period *τ*_refr_ = 2 msec is implemented.

**Synaptic Model (STDP)**

It is the well-established phenomenological model from Markram et al. [28, 36] for short-term synaptic plasticity. In the following, we refer to this model as the Markram-Tsodyks model. This model describes the effects of action potentials on the collective utilization of synaptic efficacy *u*(*t*) and the subsequent process of recovery *r*(*t*). It is an integrative model that describes both synaptic actions of depression and facilitation. It reads [3]

(2) $$\begin{array}{c} \frac{dr\left(t\right)}{dt}=\frac{1-r\left(t\right)}{\tau_{\text{rec}}}-u\left(t\right)\cdot r\left(t\right)\cdot \delta \left(t-t_{i}\right) \end{array},$$

(3) $$\begin{array}{c} \frac{du\left(t\right)}{dt}=\frac{U_{\text{SE}}-u\left(t\right)}{\tau_{\text{fac}}}+U_{\text{SE}}\cdot \left(1-u\left(t\right)\right)\cdot \delta \left(t-t_{i}\right) \end{array},$$

where *τ*_rec_ is the pool recovery time constant. *δ*(*t* − *t*_*i*_) is the Dirac delta function and represents an incoming spike at *t*_*i*_.

**Figure 1:**
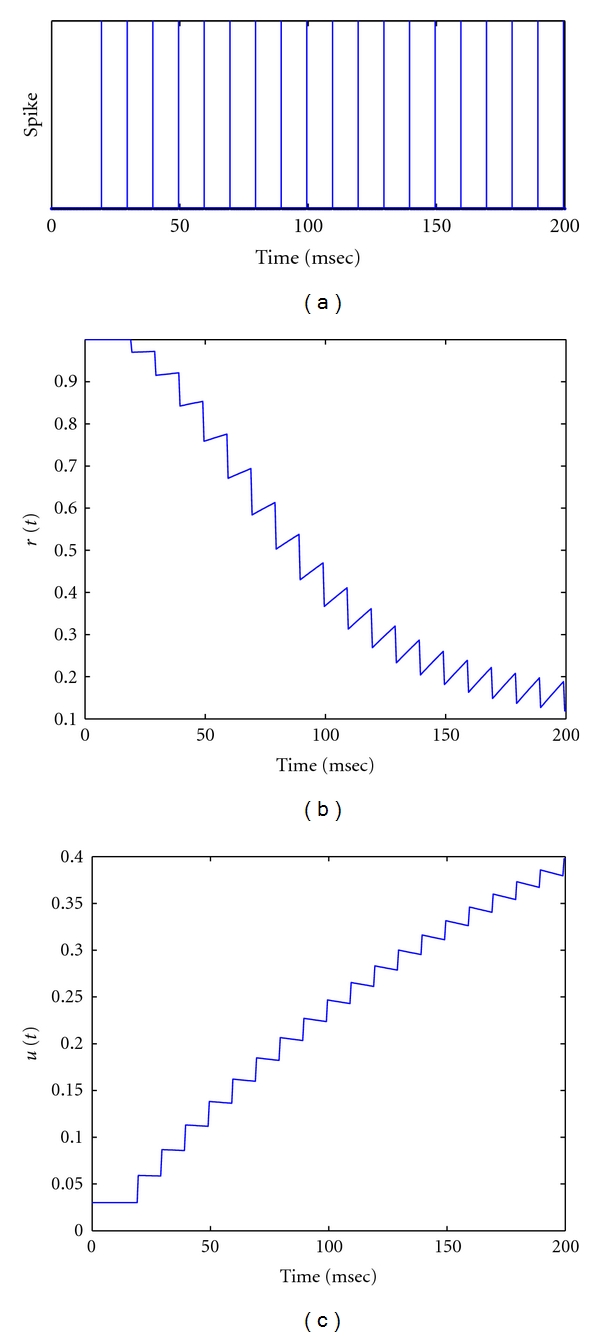
Simulating state parameters of Markram-Tsodyks model, where *τ*_rec_ = 130 msec and *τ*_fac_ = 0.5/130 msec. (a) Regular spike train stimulus at 100 Hz. Both (b) and (c) illustrate the time course of *r*(*t*) and *u*(*t*) in response to the regular spike train, respectively.

Assuming a presynaptic action potential at time *t*_*i*_, the depression process can be expressed by (2), in which *r* is the fraction of neurotransmitter pool available for transmission, *u* is the fraction of *r* to be utilized due to each spike, and it models the neurotransmitter release probability. The facilitation mechanism, on the other hand, is caused by an increase in the synaptic utilization at each presynaptic spike and can be formulated by (3). *U*_SE_ is a constant value determining the step increase in *u* and *τ*_fac_ is the relaxation time constant, where *U*_SE_ should be bounded to [0,1]. Right after an incoming spike, *u* is increased from its current value, *u*(*t*), to *u*(*t* ^+^) = *u*(*t*) + *U*_SE_ · (1 − *u*(*t*)) and drifts towards its baseline value *U*_SE_ with a time constant *τ*_fac_ between action potentials. The rule keeps *u*(*t*) < 1.  Figure 1 illustrates the response of the state parameters *r* and *u* to a regular input spike train as in Figure 1(a). The excitatory postsynaptic response (EPSP) from an action potential is obtained by EPSP(*t*) = *A* · *u*(*t*) · *r*(*t*), where *A* is the baseline level of synaptic output. In case of an inhibitory synapse, *A* → −*A*.

In this synaptic model, *A* may be viewed as the synaptic weight. It represents the spike-timing *independent* contribution in the synaptic response. The dynamic synaptic contribution *S* at any time instant *t* is evaluated as *S*(*t*) = *r*(*t*) · *u*(*t*) [26]. The value of this dynamic contribution depends on the values of the involved parameters: *U*_SE_, *τ*_fac_, and *τ*_rec_. In the next section, we explain how the learning rule tunes only the dynamic part via modulating these parameters regulating the spike-timing-dependent response.

## 3. Reinforcement Learning Framework

**Figure 2:**
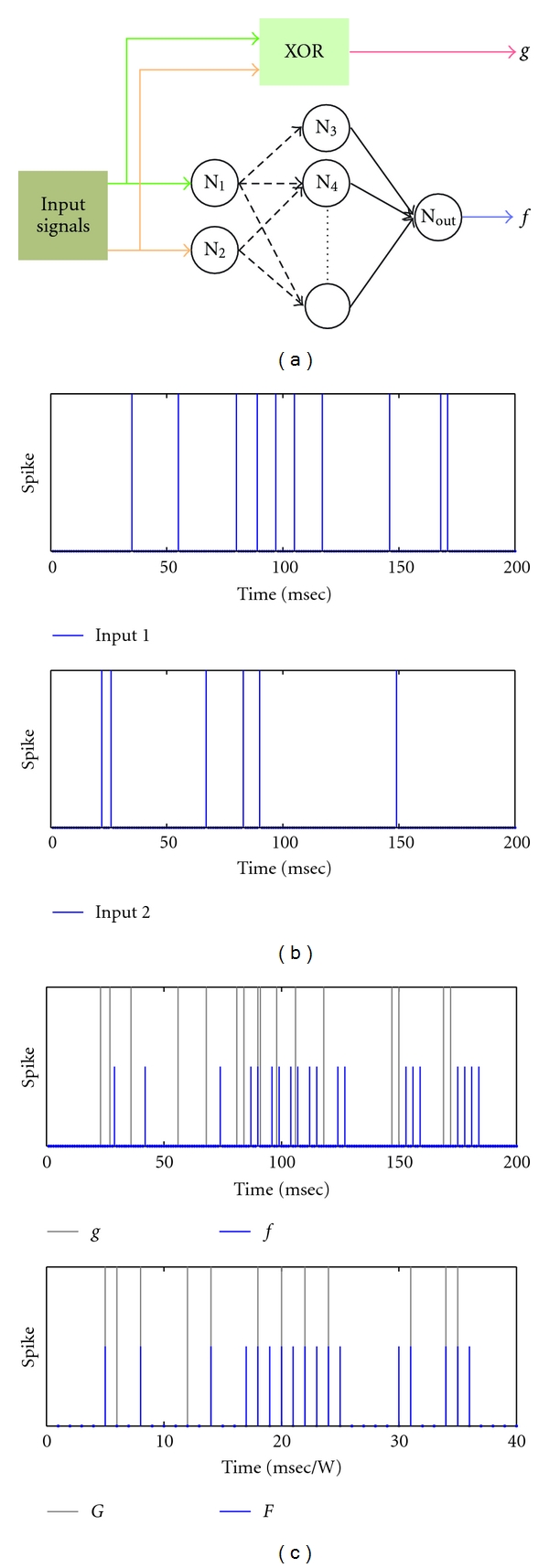
(a) Schematic representation of network setup and simulation. Different-colored input lines indicate nonidentical inputs spike trains. Dashed arrows represent those synaptic connections allowed for learning (details are explained in Section 3.4). (b) Sample of network inputs. (c) Corresponding network output. In both panels, the light gray lines indicate the locations of reference spikes *g* (or *G*). The blue lines are those correspond to *f* (or *F*). Note that in the lower panel of (c), the length of *F* (or *G*) is 200/*𝒲*, where *𝒲* = 5. Both (b) and (c) are adopted from [12].

RL is a proven tool for developing an intelligent agent without an explicit supervisor and without a teaching set, in which a reward signal is generated from the interaction with the environment, and it represents the source of supervision [34]. In order to explain the proposed learning framework, let us first consider the simulation setup. A network similar in structure to the one used in [17, 40] is considered; see Figure 2(a). The network has two input neurons *N*_1_ and *N*_2_ feeding their outputs via one hidden layer (*N*_3_, *N*_4_,…) to one output neuron *N*_out_. The network output is a spike train *f*. Inputs are spike trains with Poisson-distributed interspike intervals and are fed to input neurons. In parallel, the input spike trains are fed to an XOR gate. Details of simulation are given in Section 4. The XOR gate provides the correct output (target output) as a reference spike train *g*. A basic question is how this setup (Figure 2(a)) can be mapped to the RL configuration.

### 3.1. Reward Signal

It has been described that a reward signal (or a feedback parameter), *ℛwd*, can be derived to represent the progress in capturing certain temporal dynamics [15]. This reward is based on the difference between the target spike trains and the network's actual output. As for the distance, van Rossum introduced an algorithm, which is used here to calculate the distance between two spike trains [38]. It is a dimensionless distance that calculates the dissimilarity between two spike trains. It is calculated by filtering both trains with an exponential filter and calculating the integrated squared difference of the two trains. Each spike at time instant *t*_*j*_ in *f* is convolved with an exponential function exp⁡((*t* − *t*_*j*_)/*τ*_*c*_) with *t* > *t*_*j*_, leading to the time series *f*(*t*). Likewise, each spike in *g* is convolved with this exponential function, resulting in the time series *g*(*t*). From the resulting time series *f*(*t*) and *g*(*t*), the van Rossum distance measure reads

(4) $$\begin{array}{c} 𝒟\left(f,g\right)=\frac{1}{\tau_{c}}\overset{\infty }{\underset{0}{\int }}{\left[f\left(t\right)-g\left(t\right)\right]}^{2}dt, \end{array}$$

where *τ*_*c*_ is the time constant of the exponential filter. It controls the extent of the effect from each spike on the following spikes; that is, it determines the time scale of this distance measure. Here, *τ*_*c*_ is set arbitrarily to 15 msec.

In order to reduce the effect of the input variability on the observed performance [15], the reference spike train and network's output are temporally coded (or binned) with a nonoverlapping temporal window with width *𝒲* taken first to be five msec. During each time window, having one or more spikes is interpreted as having a digital one (high) otherwise as zero (low). Thus, for any spike train of length *L* that is binned with *𝒲* msec window, the spike trains are mapped to shorter versions with length *L*/*𝒲*. In other words, output spike train *f* with a 200 msec epoch is mapped to a binned version *F* that is 40 steps long. Similarly, *g* is mapped to *G*; see Figure 2(c). Hence, the reward signal is defined as

(5) $$\begin{array}{c} ℛwd=e^{-\alpha 𝒟(F,G)} \end{array},$$

where *α* = 0.01. This definition of *ℛwd* maps the distance *𝒟* ∈ [0, *∞*) to the range (0,1], with a maximum reward value of unity when the distance vanishes, that is, at identical outputs. *ℛwd* is dimensionless; this is a key property in the introduced framework because of the required consistency of physical units (which will be clear in (6)). The value of the reward signal is used to modulate synaptic parameters that represent certain biophysical quantities with physical units rather.

### 3.2. Mapping the Simulation Setup to an RL Scheme

In a standard RL problem, an agent represents the learner and the decision maker. Everything outside the agent is its environment. The environment tells its agent about its current state (activity), and it also gives rise to rewards. The agent tries to maximize these rewards over time [34]. As for the used temporal difference (TD) RL scheme here, the environment state is the input patterns represented during each episode (trial). The policy is formulated by both the synaptic model and the update rules, it sets the dynamic synaptic strength that is used in each trial dynamically. The action is the output spike train from the ANN (resp., from its output neuron). The XOR gate and the calculation of *𝒟* are viewed as an advisor for the learning agent. Differently stated, the network itself is the agent. This agent has two policies; they are the synaptic parametrization and the update rule. Attached to this agent, there is an advisor. The latter calculates the distance from the reference spike train, apply binning and feed the reward value to the update rule (the agent's second policy). (Two rules support this description of the RL setup [34]. First, a policy represents a sensory-output rule. It is the agent's way of behaving to the input information. Second, anything that cannot be changed arbitrarily by the agent is considered to be outside of it and thus part of its environment.) The enhancement in the synaptic model (the agent's first policy) aiming to improve the quality of the action is better derived by a temporal difference error rather than the reward values [15]. In other words, instead of modulating the changes in the parameters subject to training with the reward values, the temporal error between the desired reward and current reward values is used. This approach was basically introduced without neurobiological evidences, as it was not developed for neural networks at first place [34]. Researches in neuroscience have discovered that the firing activity of dopamine neurons in many cortical regions appear to resemble this error function in the TD algorithm [8, 32].

### 3.3. Hebbian Update Rule

The dynamics of synaptic action are governed through the contribution of electrochemical mechanisms represented via the model parameters: *U*_SE_, *τ*_rec_, and *τ*_fac_. Each of these parameters is denoted by *m*. The value of *m* is either increased, or decreased following the Hebbian rule Δ*m* = *η* · *m*, where |*η* | < 1 is the learning rate [15, 29], according to the pairing between pre- and postsynaptic activity. The realization of this basic Hebbian rule reads as follows: the values of parameters contributing to the facilitatory mechanisms are increased and the contribution of the depressive mechanisms are decreased when a spike at the presynaptic neuron induce a desired spike at the postsynaptic neuron. The term “desired” refers here to a correct hit. If the presynaptic spike does not induce a postsynaptic spike and no spike is expected the process is flipped. Whether the spike is desired or not is judged by comparing to the reference spike train. So far, it is supervised learning in full sense. For the TD learning framework, we use a reward-based error signal *δ*_*ℛwd*_ applied to the eligible synapses to update their parameters

(6) $$\Delta m=\eta \cdot m\cdot \delta_{ℛwd},$$

where *η* is set to 0.01. *δ*_*ℛwd*_ is the temporal difference error that is usually calculated as a prediction error. It is normally calculated as the difference between the ideal (or expected) reward and a scaled value of the current one [34]. Scaling the current reward is made via trace decay parameter *λ*. In this study, *δ*_*ℛwd*_ is the temporal difference error between the unscaled values (*λ* = 1) of the current reward and the previous one from the previous trial. It reads

(7) $$\delta_{ℛwd}=\mu \left({ℛwd}_{\text{previous}}-{ℛwd}_{\text{current}}\right),$$

where *μ* is a scaling factor to match the order value of *δ*_*ℛwd*_ to the order of the parameters under training. It is set to seven throughout the simulation.

On episodic basis (after each trial), the sign of the error value is used to alter the direction of the change in the parameter value, either to increase or to decrease the value of the tuned parameter. Having a signed value, this learning rule allows anti-Hebbian synaptic plasticity [15, 29]. Recalling that the direct modulation of the synaptic model parameters implement a gradient descent [1, 2], the proposed rule here optimizes the error function *δ*_*ℛwd*_ in a heuristic way. The implicit objective of achieving a stable maximum reward is preserved via minimizing the error value *δ*_*ℛwd*_ [15]. Calculating the reward values from the distance between the spike trains without binning reinforces the input variability. This deteriorates the results significantly, as the fluctuations in the temporal error will be too high. Thus, the binning is used to suppress this variability and to isolate, to a certain extent, the performance of the learning from its effect.

In this study, the hidden model parameters subject to training are *U*_SE_, *τ*_rec_, and *τ*_fac_; their initial values are arbitrarily set to 0.5, 100, and 50 msec, respectively. *A* is fixed to 7 × 10^−4^. Note that *A* in this synaptic model represents the synaptic weight. Therefore, it has been chosen in this study to be fixed and to be excluded from the training process for the sake of emphasizing the role of direct tuning of synaptic model parameters. Recalling the note mentioned above after (5) about *ℛwd* being dimensionless, if the reward values have units of, for example, bits and *m* denotes *τ*_rec_ or *τ*_fac_, (6) will not be longer correct.

**The Reference Spike Train**

In the proposed framework, the availability and need for the reference spike train represent a major issue. It may be argued that contrary to supervised learning, the actual desired output (reference) should not be used in RL to correct the behaviour of the environment. Instead, an agent extracts the required information about the next action from the history of both the environment behaviour and rewards. This is done implicitly in the proposed framework. The distance between the output and reference spike train is applied only on episodic basis. Thus, the history of the networks behaviour is used, as it is compared to the reference one and the distance gives rise to the reward signal. Therefore, the proposed framework models correctly an RL problem with a plausible realization to synaptic STDP. As mentioned, the value function here is the distance between the reference and output spike trains. From a macroscopic (cognitive) point of view, the need for the reference spike train calls for the need of memory to accomplish learning in general. This, in turn, raises a fundamental question of whether memory is a prerequisite for learning or not. Here, we entertain that memory is needed for learning, at least for the condition when the input information has never been presented to the network. In the simulations presented here, this condition is fulfilled.

### 3.4. Eligibility Traces

Eligibility denotes synapses that have contributed to either a correct or false output spike. These eligible synapses can be determined either analytically as in [15, 17, 40] or phenomenologically as in [16, 22, 25]. In order to keep complexity at a minimum, the latter approach is the one adopted in the presented study. In general, this approach depends on the understanding of the flow of spiking activities within the network. In other words, for a series of neuronal activities, synapses of the neural network do not influence the timing of the output spike with identical contributions. In the study at hands, it is chosen to allow training for only the forward synaptic connections between the input neurons and the hidden neurons (shown as dashed lines in Figure 2(a)). That is, only the model parameters of those forward synapses are updated according to the proposed learning framework.

## 4. Simulation and Results

 The input data is a set of 600 spike trains with total epoch of 200 msec at 1 msec discretization each. Each input spike train has a Poisson distributed interspike intervals with an overall frequency of 50 Hz. This set is arranged in two subsets, each of which is the input set for one input neuron.  Figure 2(b) shows a sample of the two input spike trains, note that the epoch here is the simulation epoch of 200 msec (*L*). Samples of the output spike train and the reference one are given in Figure 2(c) as well as their corresponding binned versions.

Beside the values of the reward, the performance is demonstrated with the distance *𝒟* between the two short representations of reference and network output spike trains as well. And it is calculated per episode. Taking into account the role of the temporal features embedded in the input (and output) spike trains, other indicator of performance is considered. This indicator is the maximum cross-correlation coefficient *𝒳* between the Gaussian-filtered versions of *F* and *G*; *F* and *G* are the binned (short) versions of the output and the reference spike trains *f* and *g*, respectively. This indicator is never used in the training or in updating the values of the model parameters.

**Figure 3:**
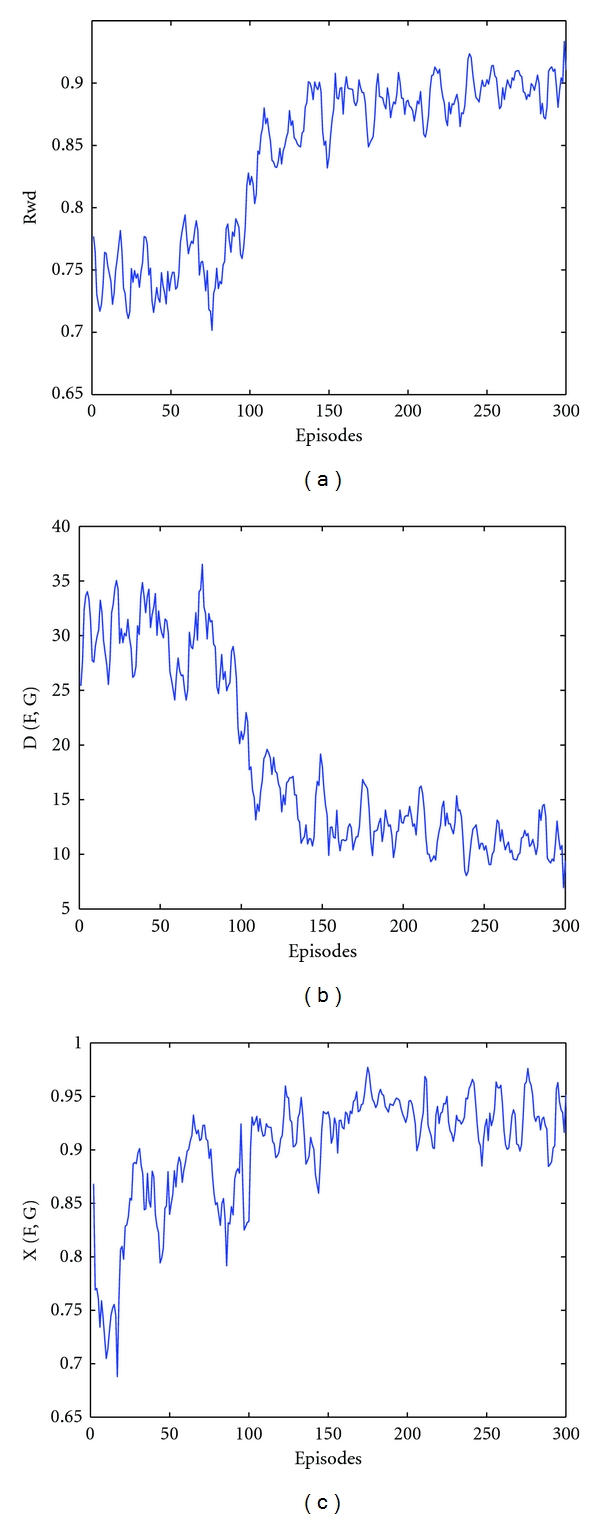
Simulation results in case of five neurons in the hidden layer and window size set to five msec. (a) Values of reward signal. (b) Distances between the reference and the output signal, *𝒟*(*F*, *G*). (c) Maximum cross-correlation coefficient observed between the reference and the output signal, *𝒳*(*F*, *G*). A snapshot from the simulation over the input/output firing patterns and internal EPSP of the output neuron is given in Figure  S1.

A network with a hidden layer of five neurons is implemented, and simulation is repeated with seven neurons in the hidden layer. The network has one output neuron; that is, the network size is *N* = seven and 10 neurons respectively. The minimum number of neurons, required to solve the XOR problem is five. In both networks, two synapses between input neurons and the hidden layer are randomly selected to be inhibitory synapses. Between the hidden layer and the output neuron, only one synapse is selected inhibitory. The selection of inhibitory synapses is not changed during the simulation. For the smaller network (*N* = 7), mean values of *𝒟*(*F*, *G*) and *𝒳*(*F*, *G*) over the last 50 episodes are 10.9 ± 1.5 and 0.83 ± 0.068, respectively. For this network, the reward signal is given in Figure 3(a), and performance measures are illustrated in Figures 3(b) and 3(c). With the larger network (*N* = 10), similar to the previous setup the values of *𝒟*(*F*, *G*) start between 25 and 35, experience an overall decay over time and reach asymptotic stability after 100 episodes of training; the mean value over the last 50 episodes in the observed distance is 3.21 ± 2.33. *𝒳*(*F*, *G*) has a mean value over the last 50 episodes of 0.93 ± 0.03. A further detailed overview of the network performance is given with a snapshot from the simulation over the input/output firing patterns and internal EPSP of the output neuron in Figure  S1 (Supplementary Materials are avaiable online at doi: 10.1155/2011/869348).

**Figure 4:**
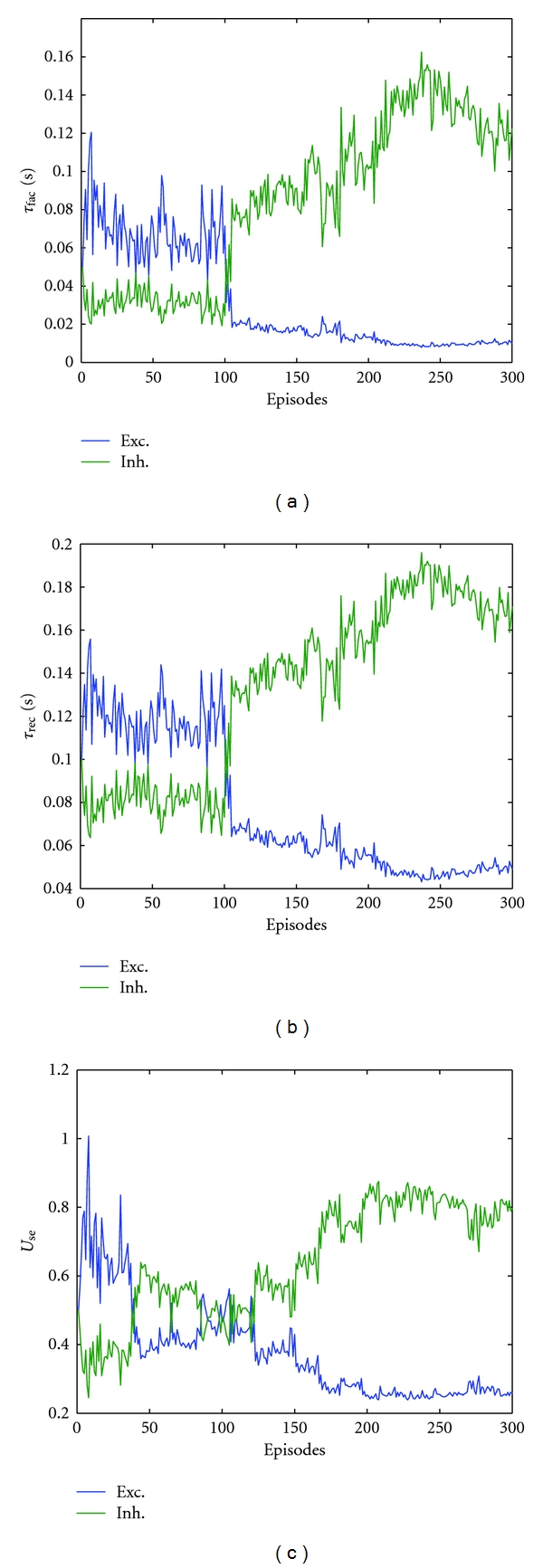
Evolution of the trained parameters: *τ*_fac_ in (a), *τ*_rec_ in (b), and *U*_SE_ in (c) over time. In all subfigures, samples from an excitatory synapse (Exc.) and from an inhibitory (Inh.) one are given. For both types, the starting values are identical.

**Figure 5:**
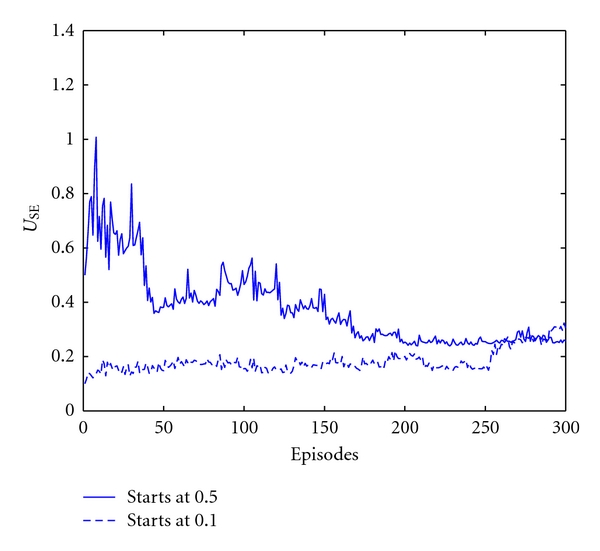
Self-organized behaviour in trained parameters. Changing the initial value of the trained parameter *U*_SE_ does not affect the final values at convergence.

The time evolutions of the trained parameters are illustrated in Figure 4. Convergence can be clearly seen from the three illustration reporting the evolution of the tuned parameters. These illustrations report the time course of the tuned parameters for both excitatory and inhibitory synapses. As for the effect of the initial values on the learning performance, different starting values are used for the parameters subject to training. Self-organized behaviour is observed. That is, the final values of trained parameters converge to self-consistent values over the training trials when either of the initial values changes.  Figure 5 illustrates an example of this for *U*_SE_, and starting the training from 0.1 instead of 0.5 leads to a similar final value at convergence.

**Figure 6:**
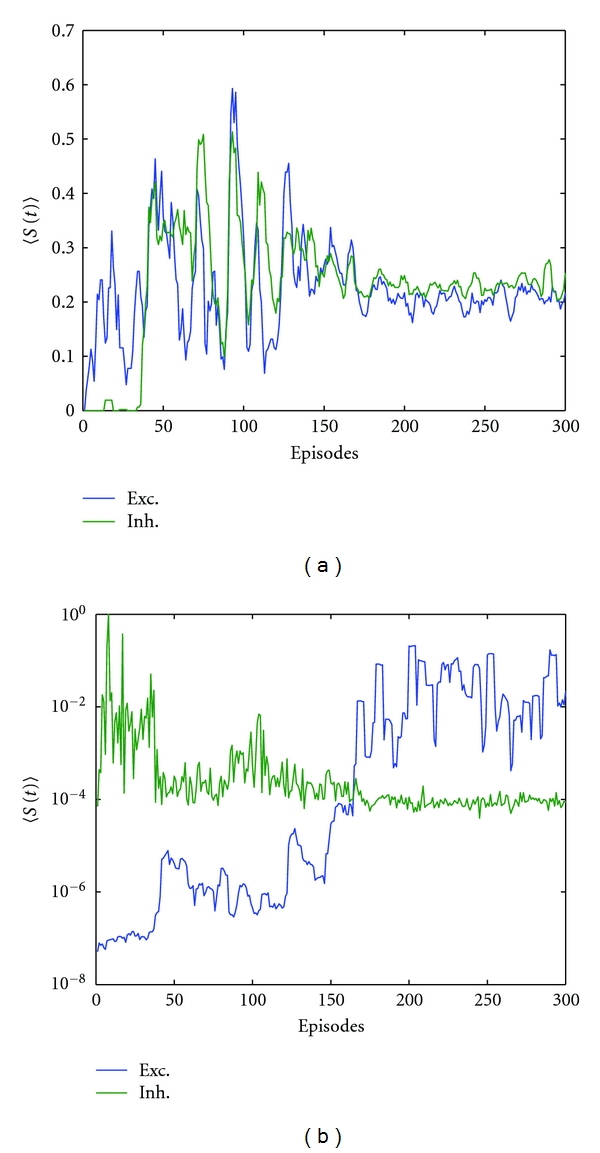
Time course of dynamic synaptic strength. 〈*S*(*t*)〉 is the time average of the synaptic strength of a synapse over all the time steps in one episode. Values are normalized between zero and unity. (a) The average dynamic synaptic strength for untrained excitatory (Exc.) and inhibitory (Inh.) synapses (smoothed). (b) The average dynamic synaptic strength for trained excitatory (Exc.) and inhibitory (Inh.) synapses (for clear illustration of the lines, *y*-axis is on a logarithmic scale).

As mentioned above, the dynamic synaptic strength of a synapse at any time instant *t* is *S*(*t*) = *r*(*t*) · *u*(*t*). Let 〈*S*(*t*)〉 be the time average of the synaptic strength of this synapse over all the time steps in one trial (episode). The time course of 〈*S*(*t*)〉 for not trained excitatory and inhibitory synapses (found between the hidden layer and the output neuron) are given in Figure 6(a); values are normalized between zero and unity and smoothed. Similarly, the time course of 〈*S*(*t*)〉 for trained excitatory and inhibitory synapses (found between the input neurons and the hidden layer) are given in Figure 6(b); values are normalized between zero and unity and *y*-axis is a logarithmic scale. By investigating the not trained time courses of dynamic synaptic strength, they almost overlap, and there are clearly two different ranges of behaviour. During the first 150 episodes, that is, before convergence, both synapses (excitatory and inhibitory) have a mean synaptic strength $\langle \hat{S}(t)\rangle$ of ≈0.233 ± 0.2. For the second half, during the last 150 episodes the mean synaptic strength is ≈0.22 ± 0.05. In case of the trained synapses, the behaviour of the synaptic strength is completely different. Both types of synapses try to optimize their ranges of influence. In other words, the excitatory synapse undergoes a progressive shift to maximize its synaptic strength and to stabilize it. Mean value increases from 2.6 × 10^−6^ ± 4.4 × 10^−6^ during the first 150 episodes up to 0.0388 ± 0.052 during the second 150 ones. The strength of the inhibitory one is lowered from 0.0126 ± 0.0833 down to 1.006 × 10^−4^ ± 4.03 × 10^−5^ and kept stable at the lowest possible range. Because of the wide span of values in the trained case, the values are shown on a semilog plot of the y-axis to clarify the differences between the two lines, see Figure 6(b).

Relative larger networks with 13, 17 and 20 neurons in hidden layer (*N* = 16, 20 and 23, resp.,) are investigated. The enhancement in the performance is observed in terms of *𝒳*(*F*, *G*) to be with an overall improvement of 0.01, 0.013, and 0.017, respectively. The effect of changing the time window is also investigated. The mean of performance measures at different binning window settings (4, 5, and 7 msec) are summarized in Table 1. In case of 4 and 7 msec window, the epoch of the input spike trains are changed in order to get a final binned version of 40 steps long; that is, the length of the input spike train is changed to be 160 and 280 msec, respectively.

The normal evaluation of results, by counting the correct hits of ones and zeros as in [17, 40] reveals relatively poor performance in the case presented here. The mean correct hit rate between *f* and *g* is 72.5% ± 1.1, while it increases to 85.4% ± 1.6 between *F* and *G* at *𝒲* = 5. It should be pointed out that, on one hand, this classical evaluation method of the results seems from our point of view not applicable here. The timing of occurrence of input spikes is solely the input feature to the network, because both neuronal and synaptic representation here implement temporal dynamics. Comparing only the counts (hit rates) of the occurrences of ones and zeros in the output and reference spike trains suppresses all the temporal information and eliminates the involvement of the STDP realized by the synaptic dynamics. Which is why we use the distance between the two-binned spike trains and the maximum coefficient of cross-correlation between them as indicators for performance. Both measures are sensitive to temporal information within spike trains. On the other hand, the proposed framework here outperforms previous approaches from [15, 40] in terms of the needed network size, learning speed and time-to-convergence. The proposed framework with a network size of 30 neurons results in a correct hit rate of *∼*91% between *F* and *G* at *𝒲* = 5 which is still comparable to those results from [17, 40] with a doubled network size. The learning model proposed in [33, 40] is not applicable to recently developed synaptic models such as the modified stochastic synaptic model [14] or the kinetic synaptic model [24]. In the analytical derivation of these models, it was assumed that the spike generation and the utilization of synaptic resources are conditionally independent of each other. Although this is not wrong in principle, it limits the applicability of these approaches to other synaptic models that do not satisfy this condition. The proposed RL framework avoids this setback and therefore it may applied to a wide class of synaptic models.

Values of *𝒟*(·) depend on the time scale parameter *τ*_*c*_. It can be shown that the change in distance due to spike insertion and displacement is inversely proportional to *τ*_*c*_ [38]. In simple words, greater values of *τ*_*c*_ give rise to smaller values of distance between the spike trains. Since the distance measure plays a critical role in the introduced framework, the effect of *τ*_*c*_ on the performance is investigated. The simulations are repeated with values of *τ*_*c*_: 5, 10, 15, 20 and 25 msec with five neurons in the hidden layer and at *𝒲* at five msec. As long as *τ*_*c*_ > *τ*_refr_ and *τ*_*c*_ ≪ *L*, no significant influence on the performance is observed. Otherwise, that is, either at *τ*_*c*_ < 7 msec or *τ*_*c*_ > 25 msec in the proposed setup, the reward values *δ*_*ℛwd*_ are too large (or too small) to correct the direction and the update rate properly. Therefore, the performance turns to be critically stable. This can be compensated by changing the scaling factor *μ* correspondingly. Similar limitation was reported in [15] as the Gaussian filtered version were used instead of the exponential ones as smoothing filters for the spike trains. The restrictions made on *τ*_*c*_ here do not limit the usage of the learning framework.

The values of *α* and *μ* are relatively related. *α* adjusts the range of the reward signal. Specifically, it adjust the minimum and the maximum values of the reward values between the zero and one depending on the span of the distance values. Corresponding to this range, *μ* either amplifies or reduces the effect of the *δ*_*ℛwd*_ on the learning rate *η*. Thus, the overall performance can be slightly sensitive to certain combinations of *α* and *μ*. This sensitivity is, however, changes depending on the input data set because the key player is the range of the distance *𝒟* values. For example, when the distances between the network responses and their corresponding reference spike trains vary between 5 and 30, the values of *α* and *μ* are chosen as reported in the script. When these distances vary between 10 and 150, *α* and *μ* should be differently selected. In general *α* is selected to make the range of *ℛwd* closer to unity. Other values are to be adjusted accordingly.

## 5. Discussion

Developing this framework is basically motivated by the need of a proper and simple learning algorithm for the spiking networks that utilize dynamic synapses. In these networks, the synapses are not represented as weighting constants. Hence, altering the synaptic response via the classical backpropagation or the *δ*-rule is not appropriate [17]. Moreover, the analytical derivation, for example in [40] and other similar studies are based, to a certain extent, on the assumption that the neurotransmitter release is independent of the spike generation process at any particular time. Although this is not wrong as an assumption, it limits the application of their techniques from being extended for other synaptic models, in which the probabilistic nature of the neurotransmitter release is only responsible for the spike generation [13, 24].

In the study at hands, a framework is proposed to direct the tuning process to the hidden synaptic parameters instead of the scaling synaptic weight. Investigating the behaviour of the synaptic dynamic strength from Figure 6 gives more insight into the influence of the learning framework proposed here. As seen from the time evolution of the synaptic strength in case of the untrained synapses, only the range of fluctuation is affected by convergence. For the trained synapses, the synaptic strength shifts to a completely new dynamic regime at convergence. This is valid for both the excitatory and inhibitory synapses. Apart from the exact numerical values, this behaviour indicates that the learning framework proposed here is able to regulate the dynamic part of the synaptic response and to capture the required input-output relation.

As for the XOR computations, comparing our results to the count of correct hits reported for example in [17] may be performed in a further study. Accounting for the temporal features in the output is a key issue that distinguishes the framework presented in this study from former ones. The output of the network here is highly characterized by its temporal contents. A distance measure that accounts for the statistical features of the compared signals, for example, stochastic event synchrony measure [10], may represent an added value to the represented framework. Determining which distance measure to use is a research point to be tackled in a future study.

The detected self-organizing behaviour for the tuned parameters suggests that the synaptic dynamics encode the statistical features of the interspike intervals implicitly. In other words, the temporal information embedded within input spike trains are encoded in the dynamics of the synaptic connections. This demonstrates the central role of the implemented learning framework not only in realizing the required computation, but also to capture the input temporal information and to store it within the synaptic dynamics.

The availability of the reference spike train along with holding the reward value from previous trial require a memory in the simulated neural system. This does not represent a problem in simulation environments, since computers are equipped with enough computational resources to accomplish this. However, this raises an important issue when the biological counterparts are under investigation. Are biological neural systems able to provide such reference outputs and keep some kind of traces to indicate the previous success (or failure) in generating their recent outputs? There is no means to check whether there is an ability to generate a reference output; however, there are evidences that neural systems keep traces about the correctness of the recent neural actions; the detection of the so called P300 signal in brain-computer interface experiments is a direct example of such indication; see, for example, [35] for a recent review. This signal is a specific form of electroencephalogram (EEG) waves and is used as a measure of cognitive function in decision-making processes. The mechanism underlying the generation of such signal is not clear, and their existence suggests, however, that neural systems compare their planned response to the required, that is, correct, response. This sheds some light on the plausibility of the proposed framework.

Intuitively, the introduced framework is not confined to the Markram-Tsodyks synaptic model. It is applicable to a wide range of dynamic synaptic models that satisfy the main assumption of underlying finite-impulse response dynamics; see, for example, [24]. Besides, the estimation of the reward signal does not presume certain characteristics of the synaptic model. Based on this sense of generality, the approach presented here is useful in cases where stochastic, biologically plausible or complex representations are required in the simulation [13, 14]. Being able to capture the XOR computation supports using this approach for tasks that require intuitively signal processing and computational capabilities. Considering the simple mathematical implementation of the update rule and calculation of the reward values, this framework can be used as an online adaptive scheme for controlling and tuning networks performance.

This study is an introductory case to be followed in order to extend the presented approach and to investigate it with larger networks that may comprise multihidden layers. Besides, it is to be tested in achieving more complex tasks than the XOR problem. Also, the use of other neuronal and synaptic representations still represents a coming task to be considered. Besides, the algorithm has not been proven to be optimal in the sense of learning speed or convergence to minimal error, it may be amenable to improvements. The stability analysis represents an open question as well; this analysis should be directly related to the adopted synaptic model as well as to the value function for the reward signal.

## 6. Conclusion

In this study, a learning framework is presented. It is based on the Hebbian/anti-Hebbian concepts of updating the values of the parameters affecting the synaptic dynamics. It is controlled via an episodic reward signal derived from the comparison between the outputs of the network and reference spike trains. The network with its synaptic dynamics are able, through the introduced learning algorithm, to implement the required nonlinear function of the XOR computations. By entertaining the hypothesis that certain mechanisms within biological neural systems may be viewed as learning via rewarding [8, 9] the biological plausibility of the approach is a main aspect in this study considering machine learning as a main target. In other words, and within the class of error-driven learning models that have some probability of being neurobiologically relevant, the proposed approach presents an alternative to classical approach of applying reinforcement learning to modulate synaptic weights. As such, it brings models for reinforcement learning closer to plausible models of unsupervised learning while realizing the Hebbian perspectives. Follow-up studies are planned to investigate the learning performance of the introduced framework with other synaptic models. Moreover, it remains to be seen if the introduced framework might be used to extend the storage capacity of a network in terms of the number of input patterns that can be stored and retrieved. 

## Supplementary Material

This section provides an extra snapshot over the internal dynamics of the network. The presented information illustrates the actual network activity in response to the input signals. The activity of the network is demonstrated at a specific time instant in terms of the EPSP of the output neuron, the firing behaviour of the three neurons and the short binned versions of the output/reference signals.
The EPSP of the output neuron reflects the spike timing dependant plasticity (STDP) discussed at the beginning of this work. It can be seen by studying the response time evolution in Figure 1(a) Supplementary, the magnitude of the excitatory response changes significantly with the change in the input spiking history as time evolves. The variability of the synaptic action in response to the inputs is characterized by the state parameters *r*(*t*) and *u*(*t*).

## Figures and Tables

**Table 1:** Summary of performance measures for the network with 7 neurons in the hidden layer.

Window	Distance	Max. cross-correl. coeff.
*𝒲*	*𝒟*(*F*, *G*)	*𝒳*(*F*, *G*)(%)
4 msec	4.63 ± 5.12	89.50 ± 6.01
5 msec	3.21 ± 2.33	93.09 ± 3.04
7 msec	6.83 ± 5.41	94.42 ± 3.01
