# Supplementary figures and images for: A Reinforcement Learning Framework for Spiking Networks with Dynamic Synapses
Source: Comput Intell Neurosci. 2011 Oct 23;2011:869348. doi: 10.1155/2011/869348 (PMC3204373; doi:10.1155/2011/869348)

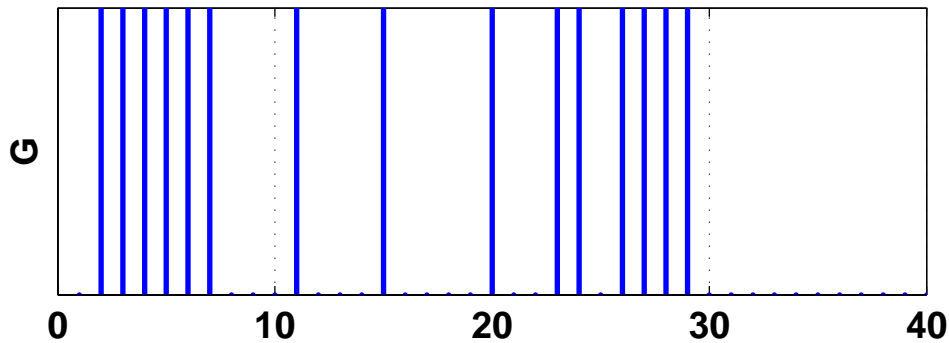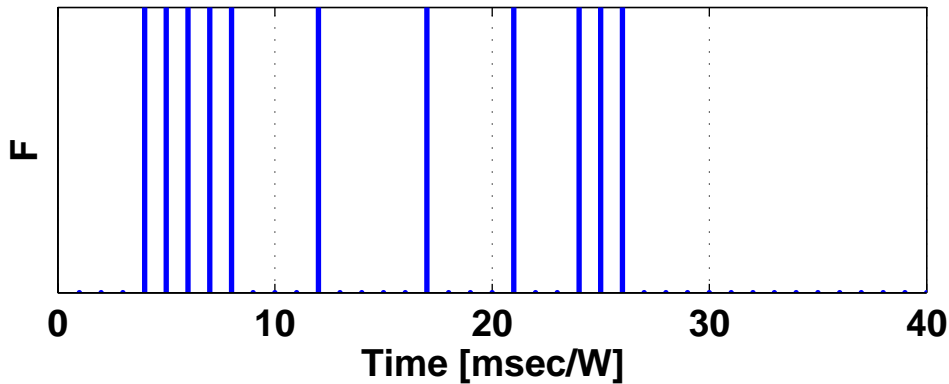

Supplement: Supplementary file 1 — This section provides an extra snapshot over the internal dynamics of the network. The presented information illustrates the actual network activity in response to the input signals. The activity of the network is demonstrated at a specific time instant in terms of the EPSP of the output neuron, the firing behaviour of the three neurons and the short binned versions of the output/reference signals. The EPSP of the output neuron reflects the spike timing dependant plasticity (STDP) discussed at the beginning of this work. It can be seen by studying the response time evolution in Figure 1(a) Supplementary, the magnitude of the excitatory response changes significantly with the change in the input spiking history as time evolves. The variability of the synaptic action in response to the inputs is characterized by the state parameters r(t) and u(t). [file 869348.f1.pdf]
